# Differential Effect of Artemisinin Against Cancer Cell Lines

**DOI:** 10.1007/s13659-014-0024-4

**Published:** 2014-06-05

**Authors:** Mounir Tilaoui, Hassan Ait Mouse, Abdeslam Jaafari, Abdelmajid Zyad

**Affiliations:** Laboratory of Biological Engineering, Natural Substances, Cellular and Molecular Immuno-pharmacology, Immunobiology of Cancer Cells Cluster, Faculty of Science and Technology, P. Box 523, 23000 Béni-Mellal, Morocco

**Keywords:** Artemisinin, Cytotoxicity, Apoptosis/necrosis, Synergism, Antitumor activity

## Abstract

The present study aims at defining the differential cytotoxicity effect of artemisinin toward P815 (murin mastocytoma) and BSR (kidney adenocarcinoma of hamster) cell lines. Cytotoxicity was measured by the growth inhibition using MTT assay. These in vitro cytotoxicity studies were complemented by the determination of apoptotic DNA fragmentation and Annexin V- streptavidin-FITC assay. Furthermore, we examined the in vitro synergism between artemisinin and the chemotherapeutic drug, vincristin. The in vivo study was investigated using the DBA2/P815 (H2d) mouse model. While artemisinin acted on both tumor cell lines, P815 was much more sensitive to this drug than BSR cells, as revealed by the respective IC_50_ values (12 µM for P815 and 52 µM for BSR cells). On another hand, and interestingly, apoptosis was induced in P815 but not induced in BSR. These data, reveal an interesting differential cytotoxic effect, suggesting the existence of different molecular interactions between artemisinin and the studied cell lines. In vivo, our results clearly showed that the oral administration of artemisinin inhibited solid tumor development. Our study demonstrates that artemisinin caused differential cytotoxic effects depending not only on the concentration and time of exposure but also on the target cells.

## Introduction

*Artemisia annua* L., a Chinese medicinal herb has evoked wide interest for its artemisinin content. This sesquiterpene lactone compound contains an endoperoxide bridge that forms a carbon-base free radical, when encountering an iron atom [1, 2]. When formed, free intracellular radicals cause molecular damages and could lead to cell death.

The artemisinin molecule contains an endoperoxide bridge (–C–O–O–C–) that interacts with Fe(II) to form free radicals [1, 2]. An intact endoperoxide is crucial, since artemisinin derivatives lacking an endoperoxide bridge are devoid of antimalarial activity [2, 3]. Unlike Fe(II), Fe(III) does not cause a reductive session of the endoperoxide. The reaction between artemisinin and Fe(III) is very slow, and the reaction products have been attributed to acid mediated heterolytic cleavage of the peroxide [4]. Because malaria parasites contain a high amount of Fe(II) in the form of heme molecules [5], artemisinin’s anti-malarial bioactivity is due to its reaction with the intra-parasitic iron source and the generation of free radicals leading to cellular destruction [2, 6].

Due to their rapid rate of division, most cancer cells have high rates of iron intake [7] and express a high cell surface concentration of transferrin receptors [8], which are involved in the transport of iron into cells. In general, the aggressiveness of tumors is positively correlated with transferrin receptor concentration of its cells. Thus, artemisinin may be selectively toxic to cancer cells because of their high iron content. Also, normal cells pick up less iron and have better intracellular regulation of iron content. Then, they are significantly less susceptible to artemisinin.

Although these results need a confirmation to use different cell lines and although the molecular mechanisms need to be investigated, artemisinin has recently been suggested to have anticancer effects [9, 10].

**Fig. 1:**
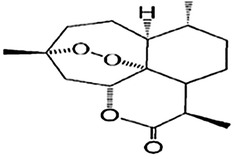
Chemical structure of artemisinin

In the present study, we report comparative data regarding the in vitro cytotoxic effect of artemisinin against tumor cell lines: P815 (murin mastocytoma) and BSR (kidney adenocarcinoma of hamster). Also, we investigate the synergistic interaction between artemisinin and vincristin against these cell lines. Furthermore, apoptosis induction in artemisinin-treated cells is investigated (Fig. 1).

## Results

### The Cytotoxicity of Artemisinin in P815 and BSR Cell Lines

**Fig. 2:**
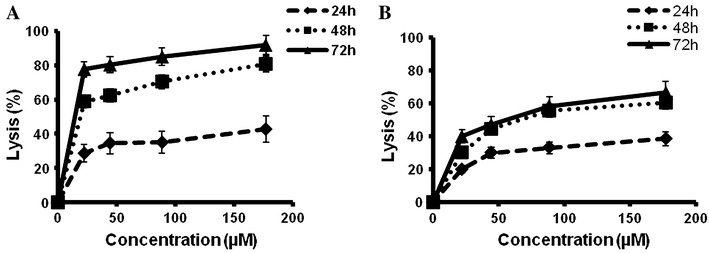
Kinetics of the in vitro cytotoxicity of artemisinin in P815 and BSR cell lines. Cells (**a** P815 and **b** BSR) were treated with increasing concentrations of artemisinin. After 24, 48, and 72 h of incubation, cytotoxicity was determined as described in Materials and methods. Each point represents the mean ± SD of three independent experiments

The in vitro cytotoxic activity was evaluated in P815 and BSR tumor cell lines. This activity is depending on the dose and time of exposure (Fig. 2).

**Fig. 3:**
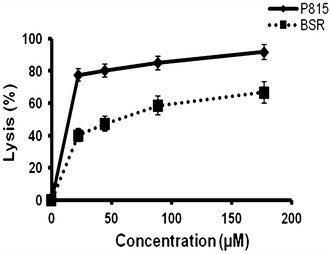
In vitro cytotoxicity of artemisinin on tumor cell lines. Cells were treated with increasing concentrations of artemisinin. After 72 h of incubation, cytotoxicity was determined as described in Materials and methods. Each point represents the mean ± SD of three independent experiments

The maximum cytotoxicity levels were obtained after an incubation time equal to 72 h. Thus, in all the following experiments, the incubation time was fixed at 72 h. The highest tested concentration had an acute cytotoxic effect reaching 90 % proliferation inhibition in P815 cells and a partial effect (about 65 %) in BSR cells. These cell lines present different degrees of sensitivity to artemisinin. In fact, the concentrations leading to 50 % cytotoxicity (IC_50_) were about 12 and 52 µM for P815 and BSR cell lines, respectively. The IC_50_ values indicate that the P815 cells are more sensitive to artemisinin treatment than the BSR cells (Fig. 3).

### Artemisinin Induced Apoptosis in P815 Cell Line

**Fig. 4:**
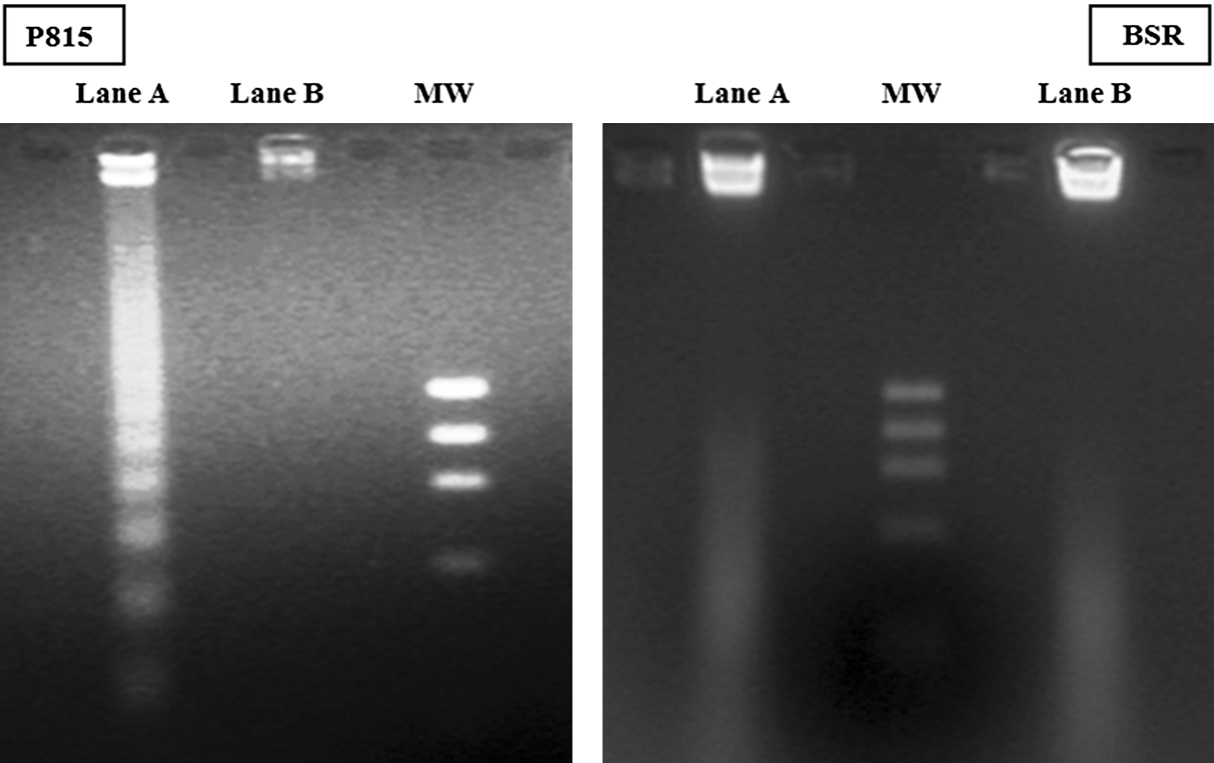
Agarose gel electrophoresis demonstrating apoptotic DNA fragmentation in P815 cells treated with 70 µM of artemisinin for 24 h. *Lane A* treatment with artemesinin 70 µM; *Lane B* control; *MW* DNA marker treatment (180–200 bp)

The apoptotic DNA fragmentation was observed in P815 cells treated with 70 µM of artemisinin for 24 h (Fig. 4, lane A). Interestingly, at the same concentration (70 µM), artemisinin did not induce DNA fragmentation in BSR cell line (Fig. 4, lane A).

#### Apoptosis Detection by Annexin V- Streptavidin-FITC Staining

**Fig. 5:**
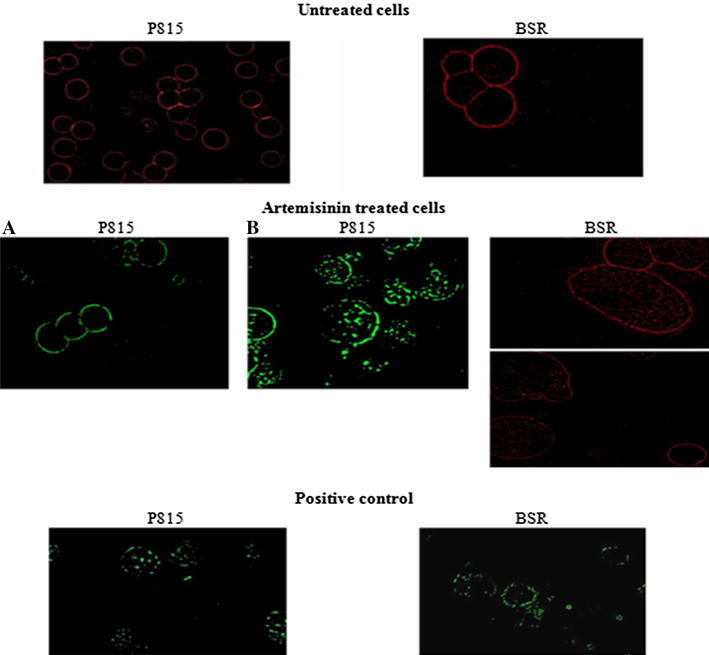
Apoptosis induction following treatment of P815 and BSR cells with artemisinin (70 µM). Untreated cells or artemisinin treated-cells were mixed with annexin V-biotin and treated sequentially with streptavidin conjugated to FITC as described in materials and methods. Cells were visualized with a microscope equipped with fluorescence filter. **a** early apoptotic cells, **b** advanced apoptotic cells. Positive control: cells cultured under conditions of serum starvation

Treatment with artemisinin (70 µM) increased the percentage of annexin V positive cells in P815 cell lines (99.2 ± 0.85 %). During the experiment, only 2.00 ± 1.25 % of the untreated P815 cells were annexin V-positive at 24 h (Fig. 5). On the other hand, BSR cells treated with artemisinin at 70 µM, showed a very low percentage of annexin V positive cells (19 ± 1.2 %) compared to positive control (82 ± 2 % of positive annexin V cells). Furthermore, a size and volume increase is clearly observed in BSR cells treated with artemisinin but not in P815 cells (Fig. 5). These results indicate that the induction of apoptosis by artemisinin is a dependent target cell.

### Cytotoxic Effect of the Combination of Artemisinin and Vincristin on P815 and BSR Cell Lines

In order to determine if artemisinin and vincristin combination display a synergistic cytotoxic activity against P815 and BSR cells, the combination index analysis (CI) method was used. For P815 cell line (Table 1), artemisinin concentration was fixed at the IC_30_ values (3 µM) and vincristin concentration varied from 0.42 to 54 µM. Vincristin induced an additive effect at 13.5 µM and an antagonistic effect at concentrations < 13.5 µM. Synergistic effect occurred for concentrations up to 27 µM combined with artemisinin at dose of 3 µM.

**Table 1:** CI analysis of vincristin with 3 µM of artemisinin in P815 cells

Vincristin (µM)	Artemisinin (µM)	Fa (A + V)	CI	Description^a^
54	3	1	0.59	Synergy
27	3	1	0.78	Synergy
13.5	3	0.99	1.32	Slight additive
6.75	3	0.92	1.95	Antagonism
3.37	3	0.76	1.82	Antagonism
1.68	3	0.73	2.20	Antagonism
0.84	3	0.69	3.82	Antagonism
0.42	3	0.68	4.83	Antagonism

*Fa* affected fraction, *A* artemisinin, *V* vincristin, *CI* combination index

^a^CI = 1.00, additive effect; CI < 1.00, synergistic effect; CI > 1, antagonistic effect

Using the BSR cell line, the concentration of artemisinin was fixed at 10 µM (IC_30_ values) and the concentration of vincristin varied from 0.42 to 54 µM (Table 2). At concentrations < 27 µM, the CI indicated an antagonistic interaction. However, when vincristin was added at increasing concentrations starting from 27 µM, the resulting interaction was nearly additive.

**Table 2:** CI analysis of vincristin with 10 µM of artemisinin in BSR cells

Vincristin (µM)	Artemesinin (µM)	Fa (A + V)	CI	Description^a^
54	10	0.93	1.15	Nearly additive
27	10	0.89	1.3	Nearly additive
13.5	10	0.86	1.4	Antagonism
6.75	10	0.83	1.935	Antagonism
3.37	10	0.81	2.94	Antagonism
1.68	10	0.80	4.605	Antagonism
0.84	10	0.80	7.89	Antagonism
0.42	10	0.76	9.945	Antagonism

*Fa* affected fraction, *A* artemisinin, *V* vincristin, *CI* combination index

^a^CI = 1.00, additive effect; CI < 1.00, synergistic effect; CI > 1, antagonistic effect

### Effect of Artemisinin on Tumor Growth in Mice

**Fig. 6:**
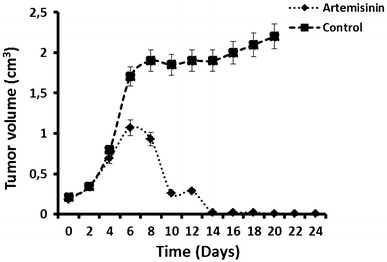
Effect of artemisinin on the evolution of tumor volume in mice

In order to evaluate if our in vitro results have a clinical relevance, artemisinin was used for in vivo assays using DBA2/P815 (H2^d^) mouse model. As shown in Fig. 6, at day zero of treatment the tumor volume was about 0.2 ± 0.06 cm^3^ for all groups tested (*P* < 0.05).

The tumor volume increase linearly and significantly in control mice between day 0 and day 6 to reach 1.7 ± 0.08 cm^3^ compared to the treated animals (1 ± 0.08 cm^3^). After the 6th day, tumor volumes of untreated mice continue to growth, which reached at day 20 a mean volume of 2.2 ± 0.1 cm^3^. Thus, tumor-bearing mice died from progressive tumors. However, in treated animals, tumor volume began to decrease to attaint 0.26 ± 0.06 cm^3^ at day 10, and close to regression (0.003 ± 0.06 cm^3^) at day 14. This difference was found to be significant between the control and treated groups (*P* < 0.05). The tumor volumes of treated animals remained constant between the 14th and 20th day, suggesting an inhibition of cell growth during this period. Our results, demonstrate that the oral administration of artemisinin reduced significantly solid tumor volume in the treated groups compared to the negative control (*P* < 0.05). In addition to increasing life span, treating tumor-bearing animals with artemisinin caused a considerable decrease in mortality percentage in comparison to non-treated tumor-bearing animals (data not shown).

Tumors were induced in DBA2 mice and treated using the protocol described in experimental section. Statistical differences between experimental groups were assessed by analysis of variance (ANOVA), with the level of significance set at *P* < 0.05.

### Effect of Artemisinin on Human Peripheral Blood Mononuclear Cells (PBMC)

**Fig. 7:**
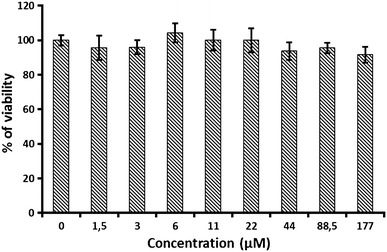
Effect of artemisinin against the PBMC

Knowing that the majority of clinically approved anticancer drugs are characterized by a narrow therapeutic window, that results mainly from a high systemic toxicity of the drugs. Thus, we tested artemisinin against the human peripheral blood mononuclear cells (PBMC), in order to determine their effects against normal cells. The results obtained are represented in Fig. 7, It is depicted that artemisinin which shows that at a concentrations able to induce a cytotoxic activity against tumor cells (P815 and BSR), no cytotoxicity effect on normal cells was observed.

PBMC were prepared from human normal donors by Ficoll-hypaque density centrifugation. Cells were incubated in 96-well microtiter plates in the presence of different concentrations of artemisinin (0–177 µM). After 48 h incubation, viability was determined using MTT assay as described in materials and methods. Data are mean ± SD of three experiments in duplicate.

## Discussion

In this paper, the in vitro antiproliferation activities of artemisinin were tested on the murin mastocytoma cell line (P815) and the kidney adinocarcinoma cell line of hamster (BSR). These activities were found to be time and dose dependent. Other investigations led to this conclusion when artemisinin cytotoxicity has been tested on the H69 human small-cell lung carcinoma (SCLC) [11].

On the other hand, we report differential cytotoxic properties of artemisinin as shown in Fig. 2. In fact, the kinetics of cytotoxicity and the IC_50_ values were dependent on the target cells. Compared to BSR cells, the P815 cell line was more sensitive to the cytotoxic activity of artemisinin (IC_50_ = 12 µM vs IC_50_ = 52 µM, respectively). Indeed the leukemic cells normally contain excess iron in their cytoplasm and the reactivity of artemisinin is enhanced by iron ions. Interestingly, normal human lymphocytes presented a weak sensitivity to artemisinin [12].

At the molecular level, artemisinins are initially activated by the cleavage of the endoperoxide with intracellular heme–iron [5, 13]. The subsequent biochemical events and cellular target(s) of artemisinin, however, remain unclear. It has been proposed that the transfer of an oxygen atom from the peroxide group of artemisinin to the chelated iron generates a Fe(IV) = O species [13, 14]. The resulting free radical intermediate may kill the target by alkylating and damaging cell proteins [13, 15].

In the present work, using two different methods, we conclude that artemisinin induced apoptosis in P815 cells and not in BSR cells. In fact, apoptotic DNA fragments were detected in agarose gel electrophoresis when P815 cells were used as target (Fig. 4). On the contrary, no apoptotic DNA fragmentation was observed in the BSR cells. These results were confirmed by Annexin V-binding assays (Fig. 5). Phosphatidylserine externalization was assessed by observing at fluorescence microscopy the extent of streptavidin-fluorescein isothiocyanate (FITC) annexin-V binding. In fact, a high percentage of annexin V positive cells was detected in P815 cells (99.2 %) but in BSR cells, there was only a poor percentage of positive cells (19 %). Interestingly, an increase in the size of artemisinin-treated BSR cells was observed. This increase is a characteristic of necrotic cell death pathway [16].

The ability of artemisinin to induce apoptosis in P815 cells is in agreement with other results reporting that artemisinin and derivatives induce apoptosis in cancer cells [17, 18]. This effect may occur during the G1 phase of the cell cycle [19]. This is understandable since enhanced transferrin receptor expression and increased iron uptake occur during this phase.

On the other hand, the evaluation of synergy or antagonism of agents used in combination is an integral part of cancer chemotherapy development. In this paper, we examined the synergistic effect of artemisinin combined with the anticancer drug vincristin, an inhibitor of cell microtubule integrity and therefore inhibitor of nucleo-spindle formation in cell division. This assay was conducted using the Chou and Talalay method [20]. These two drugs exhibited pronounced in vitro cytotoxicity when used alone against P815 and BSR cells. The synergism of vincristin (54 and 27 µM) with artemisinin at the IC_30_ values (3 µM) was observed in P815 cells. However, the antagonistic effect was shown at lower doses of vincristin. Regarding BSR cells, the interaction between vincristin (54 and 27 µM) with artemisinin at the fixed concentration of IC_30_ values (10 µM) is nearly additive; nevertheless, below the dose of 27 µM of vincristin, the antagonism occurred. The interaction between vincristin and artemisinin has not been described in the literature. To our knowledge, this is the first data about the interaction of artemisinin and vincristin; however we postulate that these results may reveal the existence of differential mechanism(s) of vincristin and artemisinin interactions in P815 and BSR cells. Furthermore, these results may have a pharmacological relevance because an anticancer drug may produce antiproliferation at low concentrations and cell death at high concentrations [21].

To find out any clinical relevance, in vivo artemisinin assays were performed in the DBA_2_/P815 (H2^d^) tumor bearing mice. We report that a regression of tumor volume was observed in artemisinin treated mice (Fig. 6). The antitumor activity of artemisinin is poorly described in the literature, although other authors have reported that daily oral intake of artemisinin could prevent or delay the development of breast cancer in the rat [10]. Indeed artemisinin decreased the number and size of tumors experimentally induced by the carcinogen (7,12-dimethylbenz[a]anthracene). Recently, Chen et al. established that dihydroartemisinin, a derivative of artemisinin, inhibited ovarian cancer cell growth in mice. In fact, at doses of 10 and 25 mg/kg respectively, this molecule resulted in 24 and 41 % tumor growth inhibition as compared to control mice, in the A2780 xenograft tumor model (*P* < 0.05), and respectively 14 and 37 % tumor growth inhibition in the OVCAR-3 model (*P* < 0.05) [22]. Furthermore, artesunate, another derivative of artemisinin, was proved to decrease tumor microvessel density and subsequently reduced tumor growth with no apparent toxicity to the animals at 50 and 100 mg/kg/day, respectively. The authors described the anti-angiogenic effect of artemisinin analogues [23–25]. Recently, Steglich et al. [26] have indicated that two artemisinin derivatives, SM616 and GHP-AJM-3/23, could be a promising P-glycoprotein inhibitor to treat cancer therapy [26].

Artemisinin may affect cancer development and growth via various mechanisms. A possible mechanism is that it selectively kills pre-cancerous cells. Artemisinin reacts with iron to form free radicals that kill cells. Various studies have suggested the involvement of iron in the development of pre-cancerous lesions. For example, iron accumulation preceded tumor formation in polycholinated biphenyl-induced liver tumor [27].

## Conclusion

In conclusion, our study demonstrates that artemisinin causes differential cytotoxic effects depending not only on the concentration and time of exposure but also on the target cells. We reported for the first time that artemisinin may induce apoptotic cell lysis depending on cancer cell type. Furthermore, we reported that the oral administration of artemisinin significantly inhibited the tumor growth of P815 in DBA2 mice. This activity was associated with an increase in life span (data not shown). On the other hand, this is the first report on the in vitro interaction between artemisinin and vincristin. Our study provides a basis for future clinical studies of artemisinin in patients with cancer, used alone or in combination with conventional anticancer drugs. An adjuvant mechanism-based therapy with artemisinin compounds may significantly improve clinical efficacy.

This research, together with the previously reported findings, will help improve our understanding about the molecular mechanisms of artemisinin and its derivatives as anticancer agents.

## Experimental Section

### Cell Culture

The cell lines (P815 and BSR) were cultured in Dulbecco’s modified Eagle’s medium (DMEM medium supplemented with 5 % heat-inactivated fetal calf serum (Gibco BRL, Cergy Pontoise, France), supplemented with penicillin G-streptomycin (1 %), and 0.2 % sodium bicarbonate (Sigma). Incubation was performed at 37 °C in a humidified atmosphere containing 5 % CO_2_.

### Cell Growth Inhibition Assay

BSR and P815 cell lines were harvested from starting cultures at the exponential growth phase. After a PBS wash, the harvested cells were poured in flat-bottomed 96-well microtiter plates containing 100 μL of complete medium per well (5 × 10^4^ cells/ml). 3 h later, several dilutions starting from the concentration 100 µM artemisinin in DMSO completed to 100 μL with complete DMEM medium were then added. Control cells were treated with DMSO alone. In all cases DMSO final concentration never exceeded 2 %. After 48 h incubation in a humidified atmosphere at 37 °C and 5 % CO_2_, 100 μL of medium was carefully removed from each well and replaced with 20 μL MTT solution (5 mg/mL PBS). After 4 h incubation under the same conditions, the cleavage of MTT to formazan by metabolically active cells was quantified by scanning the plates at 540 nm using a Multiskan EX (Finland) apparatus. Three independent sets of experiments performed in duplicate were evaluated. The relative inhibition of cell proliferation was calculated by the formula:

$$ \% {\text{ inhibition}}\; = \; 100\; \times \;\left( { 1 { } - {\text{ A}}/{\text{A}}_{\text{O}} } \right), $$

where A_O_ and A are the absorbencies of negative control and artemisinin-treated cells, respectively.

The cytotoxic effect of artemisinin against the two cell lines was compared using the IC_50_ values (artemisinin concentration leading to 50 % inhibition of cell viability).

### Kinetic Study

The cytotoxic effect of artemisinin against P815 and BSR cells was evaluated after 24, 48 and 72 h, using the MTT test in the same conditions described above.

### Apoptosis Assay

#### Fragmented DNA Electrophoresis

P815 and BSR cells (5 × 10^6^ cells) were treated with artemisinin (70 µM). After 12 h incubation in the same conditions described for cell culture, the cells were washed in PBS and treated with the lysis buffer (100 mM TRIS, 0.5 M EDTA, 10 % SDS, 5 M NaCl and 20 mg/mL Proteinase K). Samples were then incubated at 37 °C for 3 h with agitation. Then, DNA was precipitated by ispropanol and was recovered and dispersed in prelabelled Eppendorf in 10 mM Tris HCl, 0.1 mM EDTA, pH 7.5 solution. The samples of dissolved DNA were subjected to electrophoresis at 25 V for 8 h in 2 % (w/v) agarose gels complemented with ethidium bromide (1 mg/mL). Ethidium bromide-stained bands showing DNA fragmentation (180–200 bp) provide confirmation of programmed cell death. The molecular weight marker used was the λ Hae III φX 147. Separated DNA fragments (DNA ladders) were visualized using a UV trans-illuminator (310 nm).

#### Anexin V Biotin-Streptavidin FITC Test

To further confirm the apoptotic changes in P815 and BSR cells, Anexin V biotin-streptavidin FITC-stained cells were visualized with a microscope equipped with fluorescence filter (OLYMPUS OM52). Briefly, P815 and BSR cells treated with 70 µM of artemisinin or grown under conditions of serum starvation (served as a positive control) for 24 h were collected in a 15 mL centrifuge tube. After a wash in PBS, cells were stained with annexin V-biotin and treated sequentially with streptavidin conjugated to FITC. The assay is based on the ability of annexin V (green fluorescence) to bind to the phosphatidylserine exposed on the surface of cells undergoing apoptosis.

### Synergistic Studies

The degree of synergism between artemisinin and vincristin was determined by using CI analysis at a non-constant ratio, i.e., drug combinations were made by varying the concentrations of one drug (vincristin) while keeping the second drug (artemisinin) concentration fixed at IC_30_. An average CI < 1 indicates synergism, CI > 1 indicates antagonism and an average CI = 1 indicates additivity [20].

### In Vivo Antitumor Effect of Artemisinin

DBA2 mice (H2d haplotype), purchased from the animal breeding center of Orleans (France), were maintained under specific pathogen-free conditions on a 12 h light–dark cycle. Mice were provided with sterile food and water ad libitum and were used at 6–8 weeks of age with an average weight of 20–24 g. All animal experiments were performed according to national regulations which are comparable to the accepted principles for laboratory animal use and care of the European Community guidelines.

Aliquots of P815 cells (~10^7^ cells/mL) were injected subcutaneously into the left inguinal area of mice. The tumor growth and body weight of each mouse were monitored every day. Tumor volume was determined as TV (cm^3^) = L × W^2^/2, where “L” is the tumor length and “W” the tumor width [28]. Mice bearing palpable tumors were randomly divided into treatment and control groups (*n* = 6 mice/group). Artemisinin, dissolved in vegetable oil, was administered to mice of the treated group via oral route (gavage) at a dose of 80 mg/kg in a final volume of 100 µL. This quantity was administrated to mice at days 0, 2, 4, 6, 8, 10 (one oral administration every 48 h, six times). Each mice of the control group received 100 µL/2 days vegetal oil only (at the same dates as for artemisinin-treated mice). Assays were conducted in triplicate.

### Effect of Artemisinin on Human Peripheral Blood Mononuclear Cells (PBMC)

This test was realised in order to evaluate the effect of artemisinin on human normal cells using the MTT colorimetric assay described above. To isolate the PBMC, blood samples were collected from healthy human donors in heparinized tubes and the PBMC were isolated using standard Ficoll-hypaque density centrifugation. The interface lymphocytes were washed twice with phosphate-buffered solution (PBS). Cells were incubated in 96-well microtiter plates in the presence of different concentrations of artemisinin (0–177 µM).

### Statistical Analysis

The individual data values are presented as the arithmetic mean ± SD (standard deviation). The statistical significance of the results obtained from in vitro studies was evaluated by the Student’s *t* test or by ANOVA at *P* < 0.05, using STATISTICA software.
